# Des gonalgies révélatrices d'un lymphome de Burkitt

**DOI:** 10.11604/pamj.2014.18.266.5039

**Published:** 2014-08-01

**Authors:** Faida Ajili, Imene Gharsallah

**Affiliations:** 1 Service de Médecine Interne, Hôpital Militaire de Tunis, Tunisie

**Keywords:** Gonalgies, lymphome de Burkitt, lésion ostéolytique, knee pain, Burkitt lymphoma, osteolytic lesion

## Image en medicine

Le lymphome de Burkitt est un lymphome lymphoblastique B caractérisé par une prolifération monoclonale de cellules lymphoïdes B. L'atteinte osseuse au cours de ce type de lymphome est un événement rare. La localisation maxillo-faciale semble être la plus fréquente. Cependant l'atteinte des os long reste exceptionnelle. Nous rapportons une nouvelle observation d'un lymphome de Burkitt révélé par des gonalgies inflammatoires. IL s'agit d'un patient âgé de 17 ans, qui se plaint de gonalgie droite, d'allure inflammatoire évoluant depuis quelques mois. L'examen du genou ne montre pas de signes inflammatoires locaux, ni de limitation de la mobilité. La biologie trouve un SIB et une anémie à 9 g /dl. La radiographie standard du genou (A) montre la présence de lésions ostéolytiques métaphyso-épiphysaire de l'extrémité supérieure du tibia et l'imagerie par résonnance magnétique (B) une ostéolyse géographique ovalaire métaphyso-épiphysaire soufflante mesurant 59/53 cm siégeant au niveau de l'extrémité supérieure du tibia avec une zone de transition étroite qui se rehausse en péri-lésionnel sans rehaussement intra lésionnel après injection du gadolinium. La biopsie de cette lésion était non concluante et devant la survenue d'une hématémèse, une fibroscopie oeso-gastro-duodénale faite a conclu à la présence d'une antrite nodulaire et d'une lésion duodénale dont la biopsie a montré un lymphome de Burkitt. Le bilan d'extension a conclu à une atteinte ganglionnaire diffuse, pancréatique, rénale bilatérale et digestive. Le patient a été traité par chimiothérapie. L’évolution était favorable par la disparition des douleurs. Le scanner de contrôle a montré une régression des lésions osseuses (disparition des lésions tissulaires) avec début de reossification.

**Figure 1:**
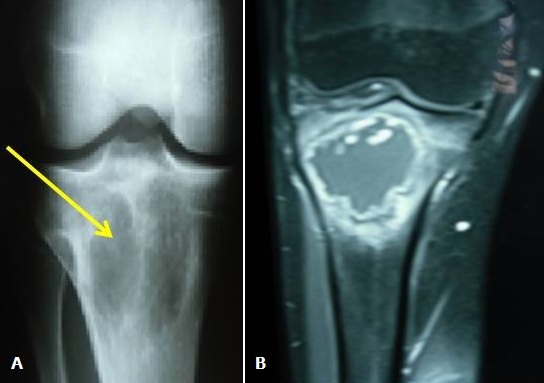
(A): Lésion ostéolytique métaphyso-épiphysaire de l'extrémité supérieure du tibia; (B): IRM du genou: ostéolyse ovalaire avec rehaussement périphérique du gadolinium

